# The Application of Multiple Strategies to Enhance Methylparaben Synthesis Using the Engineered *Saccharomyces cerevisiae*

**DOI:** 10.3390/biology14050469

**Published:** 2025-04-25

**Authors:** Lu Liu, Kai Wang, Pan Liu, Limin Ba, Huan Liu, Yanhui Liu

**Affiliations:** 1 State Key Laboratory of Green Biomanufacturing, National Energy R&D Center for Biorefinery, College of Life Science and Technology, Beijing University of Chemical Technology, Beijing 100029, China2023700073@buct.edu.cn (K.W.);; 2 Beijing Key Laboratory of Green Chemicals Biomanufacturing, Beijing Synthetic Bio-Manufacturing Technology Innovation Center, College of Life Science and Technology, Beijing University of Chemical Technology, Beijing 100029, China; 3 China Animal Husbandry Industry Co., Ltd., Beijing 100091, China

**Keywords:** methylparaben, *Saccharomyces cerevisiae*, the shikimate pathway, promoter engineering

## Abstract

Methylparaben (MP) is an important member of the paraben family of aromatic compounds, which is under great demand in the industrial market as an antibacterial agent, preservative, and feed additive, and also has potential application value in the preparation of bio-based polyetherester materials. However, the current chemical production method of MP has various problems, such as serious environmental pollution, its dependence on petrochemical resources, and the generation of different types of waste. It is of great significance to develop an environmentally friendly MP synthesis method via synthetic biology. In this work, *Saccharomyces cerevisiae* was used as the host to construct the biosynthetic pathway of MP and various metabolic engineering strategies were applied to break the bottlenecks in the synthesis process, including the regulation of the rate-limiting steps in the endogenous shikimate pathway, the enhancement of central carbon flux via knocking out competitive pathways and promoting precursors synthesis, and the improvement of the exogenous enzyme expression using promoter engineering. The final engineered *S. cerevisiae* could produce 68.59 mg/L MP in shake flasks, which was the highest titer of MP synthesized by *S. cerevisiae* so far. It was indicated that the strategies applied in our work were effective in promoting the synthesis of MP, which not only laid an important foundation for the industrial production of MP, but also provided a platform for the synthesis of other aromatic compounds.

## Simple Summary

Methylparaben (MP) is an important member of the paraben family of aromatic com-pounds. Herein, *S. cerevisiae* was selected as the chassis to achieve MP biosynthesis. The carbon flux of the targeted biosynthesis pathway was enhanced by modulating the central carbon metabolism pathways (shikimate pathway, glycolysis pathway, pentose phosphate pathway). A suitable promoter combination strategy was screened out to improve the expression of heterologous enzymes. Combined with the optimization of the fermentation process, MP was successfully produced by the engineered *S. cerevisiae*. This is the first report on MP biosynthesis using yeast and lays the foundation for further research on the bio-production of MP, as well as other parabens.

## 1. Introduction

Parabens are a widely used preservative that can be easily absorbed by the human body [1]. Their physiological safety has thus attracted much attention, and they have been reported to have extremely low toxicity and to be easily decomposed in the human body [2,3,4]. Therefore, parabens have been used in the cosmetics, food, and pharmaceutical industries. At present, parabens are allowed to be used under European Union and US legislation, while the addition of parabens in cosmetics is not restricted in Canada [5]. Methylparaben (MP) is an important paraben compound with higher safety properties than other parabens.

In the 1930s, MP was commonly used as a preservative due to its stability in a wide pH range [6]. Moreover, its antibacterial effect was better than that of sorbic acid and benzoic acid. MP was also considered to be an appropriate antibacterial agent, and was consequently widely used in various industries because of its good antibacterial effect and low toxicity. In the cosmetics industry, more than 22,000 products contain MP at a level of 0.4–0.8% [7,8]. Additionally, MP has potential application value in the preparation of bio-based polyetherester materials [9,10]. Therefore, the industrial market demand for MP has been systematically increasing in recent years due to its wide utilization worldwide.

Industrially, the synthesis of MP is mainly carried out by chemical methods via the esterification of 4-hydroxybenzoic acid (4-HBA) with excess methanol in the presence of an acid catalyst [10]. Despite the lower cost, the chemical manufacturing process not only potentially threatens human health, but also causes significant environmental pollution due to the use of strong acids and toxic organic solvents. Compared with traditional chemical synthesis methods, the synthesis of MP via engineered microorganisms is a green, safe, efficient, and sustainable process. In recent years, a few works have reported the biosynthesis of methylparaben in *Escherichia coli*. It was shown that the biosynthesis of MP could be achieved using 4-HBA as a precursor through two enzymatic reactions, namely the generation of 4-hydroxybenzoyl-CoA via the function of hydroxybenzoate CoA ligase (Hbad) and the formation of MP through catalyzing the condensation of 4-hydroxybenzoyl-CoA with the methanol by the alcohol acyltransferase (EHT1) [11]. Coupled with the enhancement of 4-HBA synthesis in the shikimate module, the final titer of MP reached 347.7 mg/L using the engineered *E. coli*. Tyrosine is an important precursor to many valuable, plant-derived chemicals, including 4-HBA. A tyrosine-linked strategy has been established in *E. coli*, *Corynebacterium glutamicum*, and *Pseudomonas putida*, using four sequential enzymatic reactions to generate 4-HBA [12,13]. Therefore, Hagel et al. [14] proposed a novel pathway to synthesize MP using tyrosine as a substrate based on a plant-derived benzoate carboxymethyltransferase (Bsmt) and four other key enzymes including phenylalanine/tyrosinase(PAL/TAL) from *Rhodosporidium cyclosporidium*, and 4-coumaroyl-CoA ligase (4CL), hydroxycinnamoyl-CoA hydratase-lyase (HCHL), and vanillin dehydratase (VDH) from *Pseudomonas putida.*, Although the titer of MP was only 201 μg/L after introducing the pathway into *E. coli*, the feasibility of the novel metabolic engineering strategy using tyrosine to produce MP was demonstrated.

**Figure 1:**
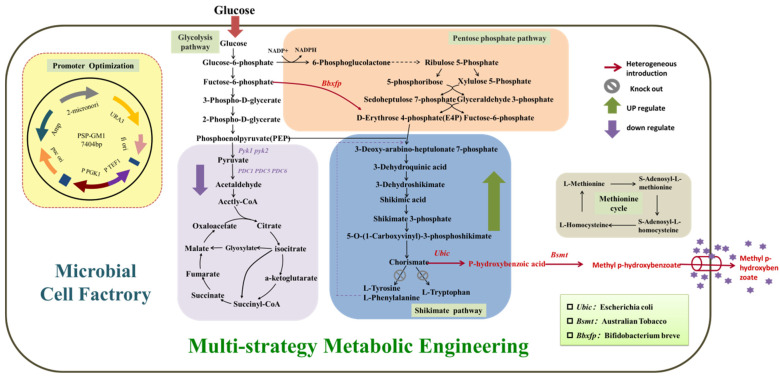
The biosynthesis pathway of MP modified in the engineered *S. cerevisiae*. Pyk, pyruvate kinase; PDC, pyruvate decarboxylase; Bbxfp, phosphoketolase; Ubic, chorismate lyase; Bsmt, benzoate carboxymethyltransferases.

While prior studies have achieved MP production in *E. coli*, this prokaryotic host has critical limitations for industrial translation, including endotoxin risks in pharmaceutical/cosmetic applications, limited compartmentalization for plant-derived enzymes like Bsmt, and weak tolerance to aromatic intermediates. At present, there is no report about MP synthesis by other microbes except *E. coli*, which limits the understanding of its biosynthesis process and the improvement of the yield. Compared with *E. coli*, the use of *Saccharomyces cerevisiae* as the commonly used eukaryotic host has preferable advantages: (1) *S. cerevisiae* is generally recognized as safe (GRAS) for food/pharma products; (2) *S. cerevisiae* has superior secretory pathways and chaperones for the functional expression of plant methyltransferases; (3) *S. cerevisiae* exhibits higher tolerance to toxic metabolites. In our work, *S. cerevisiae* was thus selected as the chassis in order to achieve MP biosynthesis. The carbon flux of the targeted biosynthesis pathway was enhanced by modulating the central carbon metabolism pathways (shikimate pathway, glycolysis pathway, pentose phosphate pathway) (Figure 1). A suitable promoter combination strategy was screened out to improve the expression of heterologous enzymes. Combined with the optimization of the fermentation process, MP was successfully produced by the engineered *S. cerevisiae*. This the first report on MP biosynthesis using yeast and lays the foundation for further research on the bio-production of MP, as well as other parabens.

## 2. Materials and Methods

### 2.1. Strains and Cultivation

*S. cerevisiae* BY4741 (MATa; ura3Δ0; Leu2Δ0; His3Δ1) was used as the chassis for the expression of MP synthesis pathway. *E. coli*-Trans10 (purchased from Quanshijin Biotechnology Co., Ltd., Beijing, China) was used for plasmid construction. All plasmids and engineered strains constructed in this work are listed in Table 1.

LB broth or an agar plate was used to cultivate *E. coli* with the addition of 100 μg/mL ampicillin. YPD medium or an agar plate (10 g/L yeast extract, 20 g/L peptone, 20 g/L glucose and 20 g/L agar) was used to cultivate original *S. cerevisiae*; selective YNB (Yeast Nitrogen Base) agar plates were used to cultivate the engineered *S. cerevisiae* which was transformed into plasmids and the positive single colonies were picked up and transferred onto the seed medium (SC-Ura/Leu, 1.7 g/L YNB, 5 g/L ammonium sulfate, 1.655 g/L amino acid mixture (lack of Ura, His, Leu), 0.86 g/L His, 0.173 g/L Leu, 20% glucose 100 mL/L, adjust pH = 6 with NaOH), which were grown at 30 °C for 12 h. Afterward, the broth was centrifuged at 4000 rpm for 2 min and the cells were collected, washed with sterile water, and prepared as suspension. After OD_600_ measurement using a spectrophotometer (Hitachi UV-2900, Tokyo, Japan), the cell suspension was inoculated into 30 mL fermentation medium in a 100 mL shake flask with an initial OD_600_ of 0.01 and was grown at 30 °C, 200 rpm for 4–5 days.

### 2.2. Plasmid Construction

The plasmids pSPGM1, pRS425, and pIYC04 were used in this work, which were stored in the laboratory. The selected chorismate lyase (*Ubic*, GenBank: CAA47181.1), from *E. coli* and the benzoate carboxymethyltransferases genes from *Australian tobacco* (*Bsmt1,* GenBank: CAF31508.1), *Arabidopsis thaliana* (*Bsmt2*, GenBank: AT3G11480), and *petunia* (*Bsmt3,* GenBank: AY233466) were codon-optimized according to the *S. cerevisiae* codon usage table, and were synthesized by the Beijing Genomics Institute. The other genes were PCR amplified from the genome of *S. cerevisiae* and *E. coli* JM109 using the primers listed in the Supplementary Data Table S1. All these genes were inserted downstream of the PGK1 and TEF1 promoters in the pSPGM1 at the *BamHI/XhoI* and *NotI/PacI* sites via T4 ligation or Gibson assembly, respectively. The constructed plasmids were verified by colony PCR and sequencing, which were then transformed into the hosts.

The CRISPR/Cas9 system was used for gene knockout. The vector PST1.G.Ura was used as the template to amplify the selectable marker using the primers containing the gRNA sequences (Supplementary Data, Table S1). And then they were inserted into the Cas9-carried plasmid Placz-Sall to form the target plasmids via Golden Gate Assembly. The donors (repair dsDNA 120bp) were obtained through the PCR amplification of two shorter oligonucleotide primers. Thereafter, the target plasmids and donors were co-transformed into yeast by electroporation.

### 2.3. Analysis Method

The target product MP was qualitatively detected by GC-MS. The fermentation broth was centrifuged to take the supernatant and was mixed with the silylation reagent BSTFA-TMCS, which was then placed at 90 °C for 30 min. After centrifuging at 12,000 rpm/min for 3 min, the supernatant were analyzed by GC-MS (Agilent 7890A Gas Chromatograph 5975MS, Palo Alto, CA, USA) equipped with the column HP-5 (30 m × 0.25 mm ID × 0.25 µm Agilent). Nitrogen was the carrier gas with a flow rate of 1.0 mL/min. The temperature of the injection block and the ion source was set up as 250 °C and 200 °C, respectively. The column temperature profile was kept at 100 °C for 4 min, followed by 30 °C/min increases up to 250 °C, and was then held at this temperature for 1 min.

For the determination of MP production, the fermentation broth was centrifuged to make 500 µL supernatant and mixed with an equal amount of ethyl acetate to extract MP. After centrifuging at 12,000 rpm/min for 3 min, 350 μL upper organic phases were taken to remove the solvent in the rotary evaporator. Afterward, 350 μL methanol was added to redissolve the product and filtered through a 0.22 μm organic membrane to prepare the samples. The production of MP was detected by HPLC equipped with a UV detector (270 nm) and Kromasil 100-5-C18 460 × 250 mm column (Nouryon, Bohus, Sweden). The column temperature was set at 30 °C and elution was performed at a flow rate of 1 mL/min with the mobile phase at 40% 0.02 mol/L ammonium acetate (A) and 60% methanol (B). The cell growth was detected by measuring the cells’ density at 600 nm (OD_600_) using a spectrophotometer (Hitachi UV-2900). All the data were obtained from at least three independent experiments for statistical analysis, and their significance was calculated using GraphPad PRISM 9 software.

## 3. Results and Discussion

### 3.1. Construction of MP Synthesis Pathway

The key enzymes in the MP synthesis path that need to be constructed are chorismate lyase and benzoate carboxymethyltransferases. Ubic encoding the chorismate lyase from *E. coli* has been proven to be effective for the accumulation of P-HBA, which was expressed in *S. cerevisiae* BY4741 to construct the synthetic pathway. Bsmt encoding the benzoate carboxymethyltransferases from plants has been proven to be effective for the accumulation of MP. The engineered strains obtained by Bsmt from Australian tobacco, Arabidopsis, and Petunia co-overexpressed with Ubic were recorded as BYL-1, BYL-2, and BYL-3, respectively.

After fermentation for 96 h, the GC-MS detection of the recombinant BYL-1, BYL-2, and BYL-3 showed that a peak with a retention time of about 5.147 min was found in the fermentation broth, which was the same as the peak time of standard substances. Further, the NIST spectral library was used to retrieve the structural formula of the compounds at peak locations, as shown in the GC-MS detection results (Supplementary data, Figure S1). The characteristic ion fragments 204 were found by comparing with the standard, and the results were searched through the NIST spectral library and compared with the structure of the standard to finally determine the retention time and mass spectrum of methylparaben and qualitatively analyze the target product.

**Figure 2:**
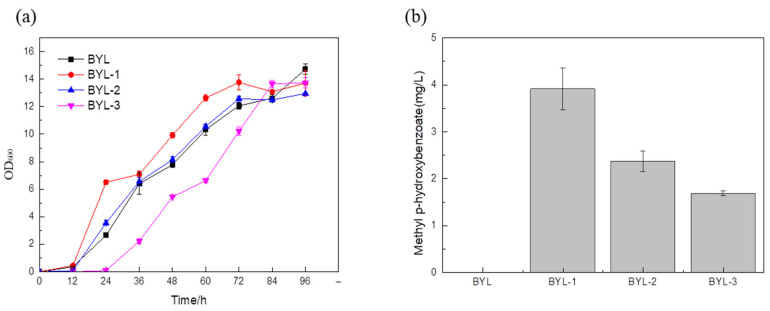
Comparison of biomass of recombinant strains. (**a**). Fermentation titer of recombinant strains. (**b**). BYL was used as the control strain. The Bmst genes of BYL-1, BYL-2, and BYL-3 were derived from *Australian tobacco*, *Arabidopsis*, and *Petunia*.

Bsmt has been proven to be effective for the synthesis of MP, and three different sources encoding the benzoate carboxymethyltransferase gene were transformed into *S. cerevisiae* BY4741 for expression to construct the MP biosynthetic pathway. The recombinant strain with the benzoate carboxymethyltransferase gene derived from *Australian tobacco* was recorded as BYL-1, and an initial MP titer of 3.9 mg/L was obtained (Figure 2).

### 3.2. Enhancement of the Carbon Flux Toward Shikimic Acid Pathway

**Figure 3:**
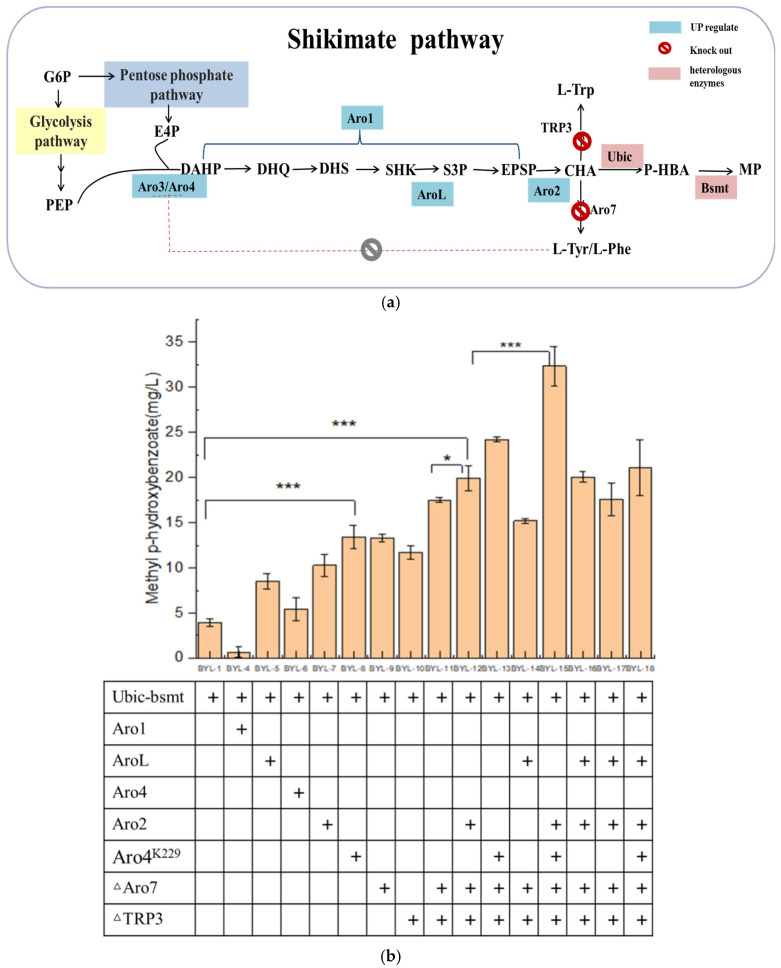
Metabolic flow chart of shikimate pathway modification. (**a**). Comparison of fermentation results of strains modified by shikimic acid pathway. (**b**). (* *p* < 0.05, *** *p* < 0.001). Abbreviations: G6P: glucose 6-phosphate, PEP: phosphoenolpyruvate, E4P: erythrose 4-phosphate, DAHP: 3-deoxy-arabino-heptulonate 7-phosphate, DHQ: 3-dehydroquinic acid, DHS: 3-dehydroshikimate, SHK: shikimate acid, S3P: shikimate 3-phosphate, EPSP: 5-O-(1-carboxyvinyl)-3-phosphoshikimate, CHA: chorismate, P-HPA: P-hydroxybenzonic acid, MP: methyl p-hydroxybenzoate, L-Trp: L-Tryptophane, L-Tyr: L-Tyrosine, L-Phe: L-Phenylalanine.

Since the carbon flux of the endogenous shikimate pathway in *Saccharomyces cerevisiae* is low, it is necessary to increase the carbon flux of the shikimate pathway (Figure 3a). Therefore, the key enzyme genes Aro4 and Aro2 (Figure 3b) of the shikimate pathway were overexpressed to obtain strains BYL-6 and BYL-7, with MP titers of 5.4 mg/L and 10.29 mg/L, respectively. The L-Tyrosine and L-Phenylalanine produced downstream of the shikimate pathway give negative feedback on the DAHP synthase encoded by the Aro4 (Figure 3b). It has been reported that the mutant Aro4(K229L) completely abolishes Tyr/Phe inhibition while maintaining >90% native activity in *S. cerevisiae*. Therefore, the mutant Aro4(K229L) is thus overexpressed to explore its effect on the synthesis of MP. The MP titer is 13.4 mg/L by the strain BYL-8 expressing Aro4(K229L), which is about 2.43 times higher than that of the control strain [15]. The introduction of shikimate kinase (encoded by AroL gene), which catalyzes shikimate (SHK) to shikimate-3-phosphate (S3P) in *E. coli*, into *Saccharomyces cerevisiae* has a certain effect on the production of aromatic amino acids. The AroL-overexpressed strain BYL-5 produced 8.509 mg/L of MP, which was about 1.06 times higher than that of the control strain BYL-1. The five-step reaction of 3-dehydroquinic acid (DHQ) to 5-enolpyruvate shikimate-3-phosphate (EPSP) is catalyzed by the pentafunctional enzyme Aro1 (Figure 3a); thus, it is speculated that this enzyme plays an important role in the shikimate pathway. For the strain BYL-4 overexpressing Aro1 gene, the obtained MP titer was 0.67 mg/L, which was due to the fact that Aro1 encodes a five-functional polypeptide, which contains similar domains of five enzymes in *E. coli* from DHQ to EPSP synthesis, whose structure is relatively large. Therefore, the Aro1 gene was constructed in the same plasmid with the Ubic and Bsmt genes at the same time, resulting in an increase in the vector burden. The gene transcription level is reduced, which affects the production of MP, or the Aro1 gene cannot be directly overexpressed in the plasmid; it may be necessary to further regulate the Aro1 gene at the genome level to increase its copy number to increase the carbon flux of the shikimate pathway.

Secondly, the biosynthetic pathway of the target product MP is based on CHA produced by the shikimate pathway as a precursor. MP is produced by introducing chorismate pyruvate lyase and benzoate carboxymethyltransferase at the same time, and the downstream pathway of chorismate mainly generates L- Aromatic amino acids such as Trp, L-Phe, and L-Tyr. To allow more carbon flux from CHA into the target product pathway, the chorismate dismutase encoded by the Aro7 gene and the anthranilate synthase encoded by the TRP3 gene were knocked out (Figure 3a). According to the fermentation results, the simultaneous knockout of Aro7 and TRP3 has a better effect on the improvement of the target product, which is consistent with the reported results. The optimal *S. cerevisiae* engineered strain BYL-15 for producing MP was determined by the optimal combination of overexpression of key enzyme genes of the shikimate pathway and the modification of the chassis cell. The titer of MP was 32.34 mg/L. Compared with BYL-11, it increased by about 84.7%, about 7.3 times higher than the original strain BYL-1, which completely proved that the enhancement of the shikimic acid pathway played an important role in the increase of the target product titer.

### 3.3. Improvement of Precursor Supply to Promote the Synthesis of MP

The production of MP is based on the endogenous shikimate pathway in *S. cerevisiae* (Figure 3a), while erythrose-4-phosphate (E4P) and phosphoenolpyruvate (PEP), as the direct precursors, significantly affect MP accumulation.

**Figure 4:**
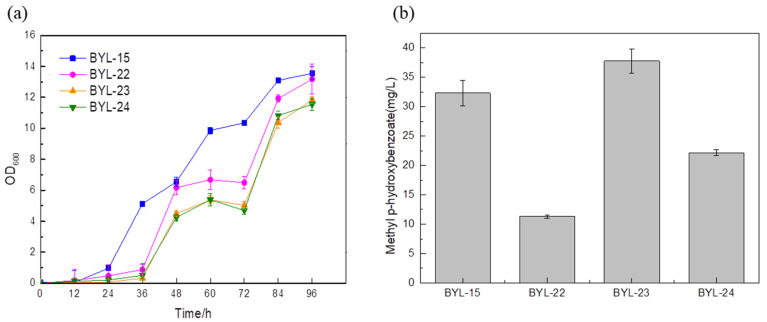
Comparison of biomass (**a**) and MP titer (**b**) of recombinant strains by improving precursor supply.

The precursor PEP of the shikimate pathway is mainly provided by the glycolytic pathway (Figure 1). Due to the Crabtree effect of *S. cerevisiae*, the pyruvate kinase converts most of the precursor PEP to pyruvate. Therefore, the main carbon flux of the PEP node is directed to pyruvate. It is then used for cell growth and ethanol fermentation, while the minor portion leads to the shikimate pathway. In the metabolic network, there are many enzymatic reactions around pyruvate and PEP, so controlling these reactions and maintaining cell growth will be an important step in the efficient synthesis of aromatic compounds [16]. In order to increase the carbon flux at the PEP node, efficiently make it lead to the shikimate pathway, and weaken the synthesis pathway from pyruvate to acetaldehyde, deficient strains were obtained by knocking out PDC1, PDC5, and PDC6 encoding pyruvate decarboxylase, respectively; these were BYL-22, BYL-23, and BYL-24. Taking strain BYL-15 as the control strain, it can be clearly observed that the biomass of the deficient strains was lower than that of the control strains, indicating that the activity of pyruvate decarboxylase was reduced. The weakened flux of the downstream energy supply module (TCA cycle) of glycolysis reduced the respiratory metabolism of cells and affected cell growth to some extent. The MP titers of strains BYL-22, BYL-23, and BYL-24 were calculated to be 11.3 mg/L, 37.75 mg/L, and 22.17 mg/L, respectively (Figure 4b). Since pyruvate decarboxylase in *Saccharomyces cerevisiae* is a protein encoded by three structural genes, PDC1, PDC5, and PDC6, in PDC1-deficient strains, in order to maintain normal cellular metabolism, the promoter on its nucleic acid sequence will regulate PDC6, and then express the fusion protein of PDC1-PDC6, but because PDC6 is a weakly expressed nucleic acid sequence, the activity of pyruvate kinase is greatly reduced [17]; according to research, it was speculated that PDC6 has the ability to guide the transport of enzymes in the metabolic process. Therefore, knocking out PDC6 will affect the related metabolic regulatory system, which in turn affects other intracellular metabolic pathways and the balance of NAD+/NADH, because NADH and NAD+ play an important role in the biosynthetic pathway and metabolic process of aromatic compounds. Therefore, PDC1 and PDC6 gene-deficient strains not only exhibit certain effects on cell growth but also lead to a serious reduction in the titer of target compounds. In addition, it was clearly observed that the strain with only PDC5 knockout had a higher titer, which was about 16.73% higher than that of the control strain. The gradual increase in the biosynthetic pathway flux of aromatic compounds and their derivatives will lead to an increase in the content of by-products such as fuel compounds, affecting the specificity of benzoate carboxymethyltransferase binding. Since PDC5 has the same catalytic ability as phenylpyruvate decarboxylase Aro10 [18], the deletion of PDC5 effectively blocked the aromatic amino acid degradation pathway, and the content of by-products was also significantly reduced, which was beneficial to the biosynthesis of methylparaben.

**Figure 5:**
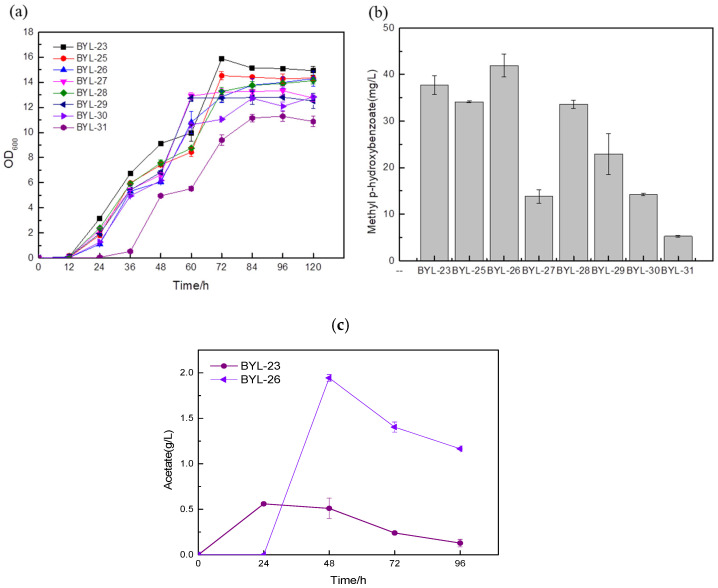
Comparison of biomass (**a**), MP titer (**b**), and acetic acid content (**c**) of recombinant strains by controlling the expression of Bbxfp with different promoters.

In *Saccharomyces cerevisiae*, about 90% of the E4P produced by the endogenous PPP pathway enters the shikimate pathway, but it is only equivalent to 8% of the PEP content that enters the shikimate pathway; according to Guo W. et al. [19], the enzyme that condenses PEP and E4P to form DAHP (DAHP synthase encoded by Aro3 and Aro4) shows a higher preference for the substrate PEP (Figure 3a). Therefore, the severe imbalance of E4P and PEP carbon fluxes into the shikimate pathway and the availability of E4P are the main problems hindering the production of aromatic compounds. In this study, borrowing from the above phosphoketolase (PHK) pathway (Figure 1), the phosphoketolase gene (*Bbxfp*) derived from *Bifidobacterium breve* was introduced, activated by seven constitutive promoters with different strengths (TEF1, TPI1, ADH1, ACS1, ALD5, CDC24, IDP1) and expressed to obtain highly expressed phosphoketolase. According to the biomass of the recombinant strains, it can be clearly observed that the growth status of the strains introduced with heterologous phosphoketolase is worse than that of the control strain BYL-23 because of a large amount of acetate produced (Figure 5a). According to the research, acetyl-phosphate (Acp) is a by-product generated when Bbxfp catalyzes F6P in *Saccharomyces cerevisiae*, which can be catalyzed to acetate by glycerophosphatase (GPP1) [20]. This conclusion was also confirmed by the detection chart of acetic acid content (Figure 5c); the accumulation of acetic acid seriously affects the growth of cells.

According to the comparative analysis of the fermentation results of the recombinant strains (b), the titers of strains BYL-30 and BYL-31 obtained by driving the Bbxfp with strong promoters TEF1p and IDP1p were 14.21 mg/L and 5.25 mg/L, respectively (Figure 5b). The titers of the strains BYL-27, BYL-28, and BYL-29 obtained by the promoters ADH1p, ACS1p, and ALD5p driving the expression of Bbxfp were 13.84 mg/L, 33.61 mg/L, and 22.9 mg/L, respectively (Figure 5b); the titer of the above five strains was significantly lower than that of the control strain BYL-23. The titer of BYL-25 and BYL-26 strains obtained by driving the expression of Bbxfp gene with weak promoters CDC24p and IDP1p were 34.11 mg/L and 41.91 mg/L, respectively (Figure 5b), and the titer of BYL-25 was about 9.63% lower than that of the control strain. The decrease in the titer of the target product may be due to the reduction in biomass, which reduces the metabolites and energy required for the synthesis pathway, and cannot achieve the growth environment required for the target product; however, the titer of strain BYL-26 is 11% higher than that of the control strain. According to this analysis, the expression of Bbxfp driven by a weak promoter has a good effect on improving the titer of the target product. This is because strong promoters and medium-strength promoters drive gene transcription at a faster rate and because the glycolytic pathway also requires F6P to provide sufficient substrates for cell growth to maintain balance throughout the entire central carbon metabolism. A weak promoter drives the expression of heterologous phosphoketolase and slows its transcription rate, effectively increasing MP production.

### 3.4. Promoter Engineering to Regulate the Expression of MP Synthesis Pathway

In synthetic biology research, promoters are the core biosynthetic elements that drive gene expression, regulate gene circuits, and obtain high enzymatic activity. Therefore, in order to achieve efficient and accurate expression of target genes, promoter engineering is one of the most critical strategies. The above experiments show that the accumulation of MP can be significantly promoted by enhancing the carbon flux of the shikimate pathway, knocking out the competitive pathway, and increasing the flux of the precursor. HBA had a high accumulation amount, but the content of the final product MP has not been greatly improved. It is speculated that Bsmt is the rate-limiting enzyme in this biosynthetic pathway. It was necessary to control the expression of Bsmt to match the supply of co-factor S-adenosyl-L-methionine (SAM). In this work, four constitutive promoters with different strengths were selected for the introduced Ubic and Bsmt, respectively, to screen for suitable promoter combination strategies.

**Figure 6:**
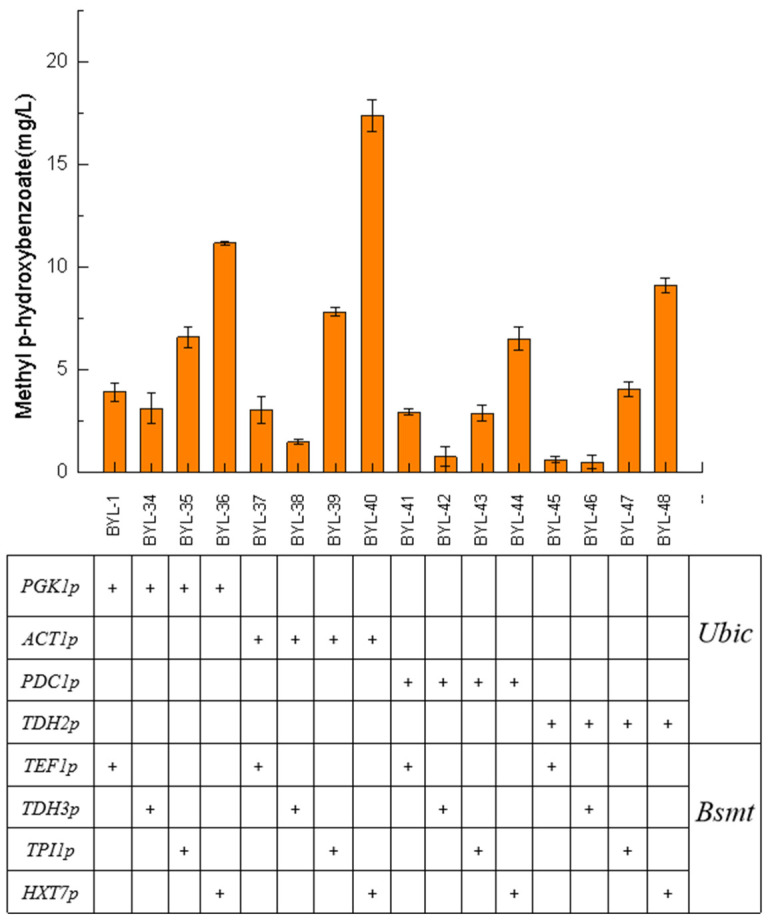
Comparison of fermentation results of recombinant strains obtained by different strength promoters driving Ubic and Bsmt gene expression.

Taking BYL-1 as the control strain, the strain BYL-40 obtained by combining the moderate-strength constitutive promoter ACT1p to activate the Ubic gene and the weak constitutive promoter HXT7p to activate the Bsmt-1 gene had the highest titer, about 17.39 mg/L, which was 3.46 times higher than that of the control strain (Figure 6). From the titer comparison of the strains with different combinations of promoters, it was obvious that the driving of the Ubic gene required a strong promoter, and as the strength of the constitutive promoter gradually weakens, the titer of the target product gradually decreases; the driving of the Bsmt-1 gene required a weaker promoter, and as the strength of the constitutive promoter gradually weakened, the titer of the target product gradually increased. CHA was converted into P-HBA under the catalysis of the enzyme encoded by *Ubic*, and the strong promoter drives the Ubic gene to make its transcription level reach a higher level. CHA was converted into P-HBA under the catalysis of the enzyme encoded by Ubic, and the strong promoter drives the Ubic gene to make its transcription level reach a high level. It can be inferred that the corresponding promoter did not drive the transcription of the Bsmt-1 gene until P-HBA reached a certain amount of accumulation. Since SAM was required to provide methyl groups in the pathway of MP synthesis, the rate of intracellular endogenous biosynthesis of SAM was slow, so the Bsmt-1 gene needed to be driven by a weak promoter to regulate the metabolic balance of intracellular substances and energy.

**Figure 7:**
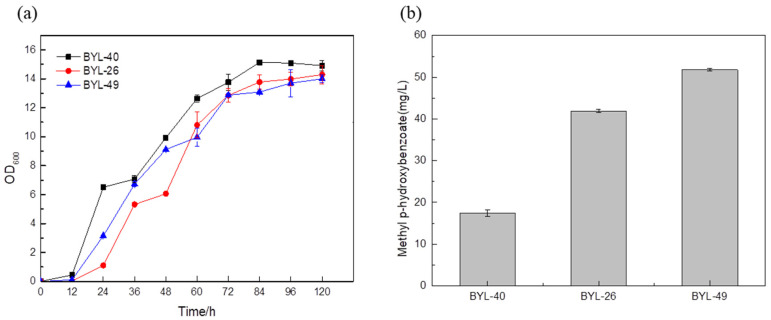
Comparison of biomass (**a**) and MP titer (**b**) of recombinant strains by combinatorial regulation of the key enzymes expression.

The MP titer reached 51.78 mg/L by the strain BYL-49 combining the optimized strain BYL-40 with the strain BYL-26 (Figure 7b), which was about 23.56% higher than that of the control strain BYL-26, thus verifying that the promoter optimization had a good effect on the titer improvement.

### 3.5. Optimization of the Fermentation Process to Accelerate the Production of MP

The composition and content of the fermentation medium have an important impact on the titer of target products [21,22]. Therefore, it is necessary to optimize the conditions and medium during the fermentation process. In order to obtain more sufficient material conditions for the optimal strain, the composition and content of the fermentation medium were explored, and the main components of the fermentation medium were screened by designing orthogonal experiments and single-factor experiments. In this way, the best medium combination can be obtained to achieve the purpose of a high titer of methyl parahydroxybenzoate.

**Figure 8:**
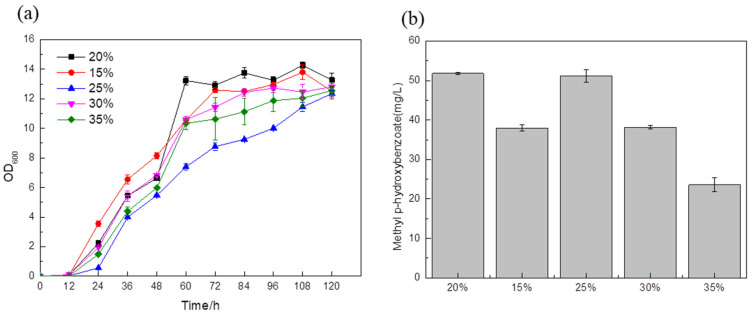
Comparison of biomass (**a**) and MP titer (**b**) of recombinant strains with different glucose contents in the medium.

The optimal strain BYL-49 obtained above was used as the strain optimized for the medium. A 20% glucose supplementation in the medium was used as a blank control. A total of 15% glucose was added to the fermentation medium, and the titer was about 38 mg/L (Figure 8b); between 0 and 48 h, the cells were in the exponential growth phase (Figure 8a), and the biomass was highest. When the target substance begins to synthesize and accumulate, the strain begins to grow slowly, which also shows that in the early stage of fermentation, 15% glucose addition can fully provide the energy and substances required for cell growth, but the rapid growth of this process may cause more carbon flux to enter the downstream energy supply module of glycolysis, resulting in an unbalanced cellular metabolism and the inability to provide sufficient a carbon source for the synthesis of target products. With the addition of 25% glucose in the fermentation medium, the titer was about 51.18 mg/L (Figure 8b), which had no obvious change with the blank control. The fermentation medium added 30% and 35% glucose while the titer was about 38.14 mg/L and 23.57 mg/L, respectively (Figure 8b). It was clearly observed that no matter whether the amount of glucose added was too high or too low, the fermentation results showed adverse effects, and the glucose content of 20% and 25% was the most suitable for the cell growth and synthesis of target products.

**Figure 9:**
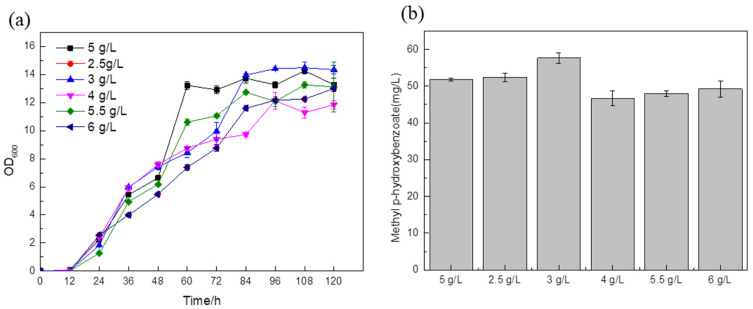
Comparison of biomass (**a**) and MP titer (**b**) of recombinant strains with different ammonium sulfate additions in the medium.

Taking the addition of 5 g/L (NH_4_)_2_SO_4_ as the blank control, according to the fermentation results, when the amounts of (NH_4_)_2_SO_4_ added were 4 g/L, 5.5 g/L, 6 g/L, the titer was slightly lower than that of the control strain (Figure 9b). The titer of the strain with 3 g/L of ammonium sulfate in the medium was 57.63 mg/L (Figure 9b), which was about 11.3% higher than that of the blank control. This shows that 3 g/L ammonium sulfate is the best condition for the growth of the *Saccharomyces cerevisiae* strain. Since nitrogen sources can adjust the pH during the fermentation process, if there are too many nitrogen sources, the bacteria will grow vigorously and the pH of the fermentation broth will be high. Since *Saccharomyces cerevisiae* prefers acidity, higher pH will affect cell growth and product accumulation; if the nitrogen source is too small, this will not only reduce the nutrients needed by the cells but may also make the medium acidic due to the fact that acidic metabolites produced during fermentation cannot reach the optimum pH for strain growth.

**Figure 10:**
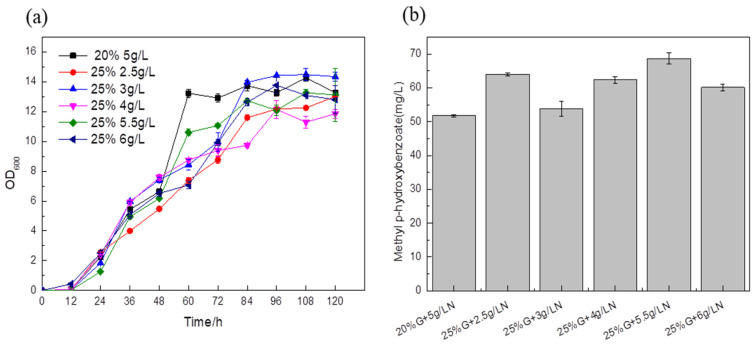
Comparison of biomass (**a**) and MP titer (**b**) of recombinant strains with different carbon–nitrogen ratios of the medium.

A total of 5 g/L ammonium sulfate and 20% glucose were added to the medium as a blank control to explore the effect of the carbon–nitrogen ratio on the titer. In the medium of the experimental group, the titers of the fermentation strains supplemented with 25% glucose and different ammonium sulfate were higher than those of the control strain, and the content of synthetic MP reached 68.59 mg/L, which was about 32.46% higher than that of the blank control (Figure 10b). This shows that an inappropriate carbon–nitrogen ratio will affect the absorption of nutrients by *Saccharomyces cerevisiae*, and directly affect the growth of *Saccharomyces cerevisiae* and the formation of products. Therefore, the optimal carbon–nitrogen ratio in the fermentation medium is beneficial to the improvement of the target product titer.

## 4. Conclusions

In this work, a heterologous pathway for the biosynthesis of methylparaben in *Saccharomyces cerevisiae* BY4741 has been successfully constructed, and various debottlenecking strategies have been investigated to enhance the ability of engineered *Saccharomyces cerevisiae* to synthesize MP. The dominant strain BYL-49 with a high titer of methylparaben was fermented in a shake flask for 120 h, and the optimal titer of MP was 68.59 mg/L. This is also the highest titer of MP biosynthesis using *Saccharomyces cerevisiae* at present, and the modified optimal strain also provides a production platform for high-yield aromatic compounds and their derivatives.

## Figures and Tables

**Table 1:** Strains and plasmids used in this study.

Strains	Description	Plasmid
BY4741	MATa; ura3Δ0; Leu2Δ0; His3Δ1	
BYL-1	Ubic(PGK1p)-Bsmt-1(TEF1p) in PSPGM1	pSP01
BYL-2	Ubic(PGK1p)-Bsmt-2(TFF1p) in PSPGM1	pSP02
BYL-3	Ubic(PGK1p)-Bsmt-3(TFF1p) in PSPGM1	pSP03
BYL-4	Aro1 in pSP01	pSP04
BYL-5	Aro2 in pSP01	pSP05
BYL-6	Aro4 in pSP01	pSP06
BYL-7	AroL in pSP01	pSP07
BYL-8	Aro4^K229^ in pSP01	pSP08
BYL-9	MATa; ura3Δ0; Leu2Δ0; His3Δ1; ΔAro7	pSP01
BYL-10	MATa; ura3Δ0; Leu2Δ0; His3Δ1; ΔTRP3	pSP01
BYL-11	MATa; ura3Δ0; Leu2Δ0; His3Δ1; ΔTRP3; ΔAro7	pSP01
BYL-12	ΔTRP3; ΔAro7; Aro2 in pSP01	pSP08
BYL-13	ΔTRP3; ΔAro7; Aro4^K229^ in pSP01	pSP05
BYL-22	BYL-15; ΔPDC1	pSP09
BYL-23	BYL-15; ΔPDC5	pSP09
BYL-24	BYL-15; ΔPDC6	pSP09
BYL-25	BYL-23; CDC24p-Bbxfp-T	PRS05
BYL-26	BYL-23; IDP1p-Bbxfp-T	PRS06
BYL-27	BYL-23; ADH1p-Bbxfp-T	PRS07
BYL-28	BYL-23; ACS1p-Bbxfp-T	PRS08
BYL-29	BYL-23; ALD5p-Bbxfp-T	PRS09
BYL-30	BYL-23; TEF1p-Bbxfp-T	PRS010
BYL-31	BYL-23; TPI1p-Bbxfp-T	PRS011
BYL-34	Ubic(PGK1p)-Bsmt-1(TDH3p) in PSPGM1	pSP011
BYL-35	Ubic(PGK1p)-Bsmt-2(TPI1p) in PSPGM1	pSP012
BYL-36	Ubic(PGK1p)-Bsmt-3(HXT7p) in PSPGM1	pSP013
BYL-37	Ubic(ACT1p)-Bsmt-3(TEF1p) in PSPGM1	pSP014
BYL-38	Ubic(ACT1p)-Bsmt-3(TDH3p) in PSPGM1	pSP015
BYL-39	Ubic(PGK1p)-Bsmt-3(TPI1p) in PSPGM1	pSP016
BYL-40	Ubic(ACT1p)-Bsmt-3(HXT7p) in PSPGM1	pSP017
BYL-41	Ubic(PDC1p)-Bsmt-1(TEF1p) in PSPGM1	pSP018
BYL-42	Ubic(PDC1p)-Bsmt-1(TDH3p) in PSPGM1	pSP019
BYL-43	Ubic(PDC1p)-Bsmt-1(TPI1p) in PSPGM1	pSP020
BYL-44	Ubic(PDC1p)-Bsmt-1(HXT7p) in PSPGM1	pSP021
BYL-45	Ubic(TDH2p)-Bsmt-1(TEF1) in PSPGM1	pSP022
BYL-46	Ubic(TDH2p)-Bsmt-1(TDH3p) in PSPGM1	pSP023
BYL-47	Ubic(TDH2p)-Bsmt-1(TPI1p) in PSPGM1	pSP024
BYL-48	Ubic(TDH2p)-Bsmt-1(HXT7p) in PSPGM1	pSP025
BYL-49	BYL-26; Ubi(ACT1p)c-Bsmt-3(HXT7p) in PSPGM1	pSP017/pRS06

## Data Availability

The data used to support the findings of this study are included within the article. Any other data can be available upon request.

## Supplementary Materials

The following supporting information can be downloaded at: https://www.mdpi.com/article/10.3390/biology14050469/s1, Figure S1: Methyl p-hydroxybenzoate GC-MS retention time (a); Results of NIST spectrum database for methyl parabens (b); Table S1: Primers and synthetic oligos used in this Study; Table S2: Comparison of MP titer and specific productivity of different engineered strains; Table S3: Genotype and copy numbers information of the plasmids used in our work.
