# Supplementary material for: The Application of Multiple Strategies to Enhance Methylparaben Synthesis Using the Engineered Saccharomyces cerevisiae
Source: Biology (Basel). 2025 Apr 25;14(5):469. doi: 10.3390/biology14050469 (PMC12108618; doi:10.3390/biology14050469)
Supplement: Supplementary file 1 [file biology-14-00469-s001.zip › biology-3568917-supplementary.pdf]

Supplementary data

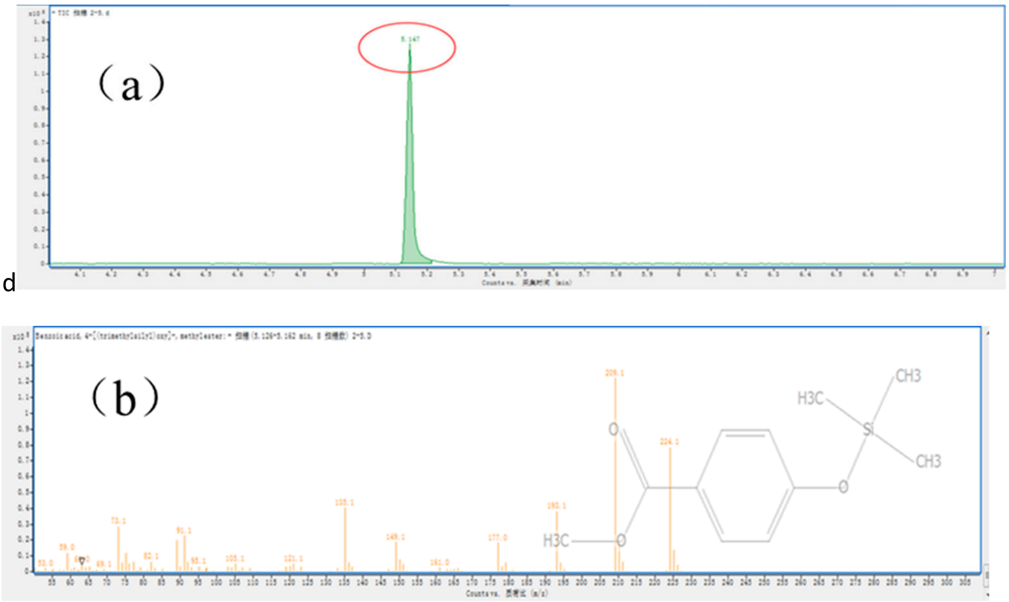

d

Figure S1 Methyl p-hydroxybenzoate GC-MS retention time (a); Results of NIST spectrum database for methyl parabens (b).

Table S1. Primers and Synthetic Oligos Used in This Study

Primer	Nucleotide sequence (5’ >3’)
Aro1-F	5’-CCTCTGGCGAAGAATTGTTACTACTCTTTCGTAACGGCATCAAAAA-3’
Aro1-R	5’-AGTTTTAATTACAAGCGGCCATGGTGCAGTTAGCCAAAGTC-3’
Aro2-F	5’-CCTCTGGCGAAGAATTGTTATTAATGAACCACGGATCTGGAGAAA-3’
Aro2-R	5’-AGTTTTAATTACAAGCGGCCATGTCAACGTTTGGGAAACTGT-3’
AroL-F	5’-CCTCTGGCGAAGAATTGTTATCAACAATTGATCGTCTGTGCCA-3’
AroL-R	5’-AGTTTTAATTACAAGCGGCCATGACACAACCTCTTTTTCTGATCG-3’
Aro4-F	5’- CGGGATCCATGAGTGAATCTCCAATGTTCG-3’
Aro4-R	5’- CCGCTCGAGCTATTTCTTGTTAACTTCTCTTCTTTGTC-3’
Aro4(k229L)-1-F	5’- CGGGATCCATGAGTGAATCTCCAATGTTCG -3’
Aro4(k229L)-1-R	5’- AGCAGCAACACCATGCAAAGTAACACCCATGAAA -3’
Aro4(k229L)-2-F	5’- CATGGTGTTGCTGCTATCACCACTA -3’
Aro4(k229L)-2-R	5’- CCGCTCGAGCTATTTCTTGTTAACTTCTCTTCTTTGTC -3’
PRS425-F	5’-TAACAATTCTTCGCCAGAGGTTTGG-3’
PRS425-R	5’GGCCGCTTGTAATTAAAACTTAGATTAGAT-3’
gRNA-Aro7-F	5’-AAAGGTCTCTGATCAAAACTCCCTTCTTTCCTGACGTTTTAG

	AGCTAGAAATAGCAAGT-3’
gRNA-Aro7-R	5’-AAAGGTCTCTAAACTAAGTGGATTCTCCTACTCAAGATCATTTATCTTTCACTGCGGAG-3’
Doner-Aro7-F	5’-TATTAACACATTACCAAATCTGTTTGGTTCTACATAGAACGCCATAAAAGTAAGAAAAAATTGAAACTTACACGGTACAA-3’
Doner-Aro7-R	5’-TGAAGGCGTTCGCCCTTACAACGTTAAGTTTTTTCCCTATTGTACCGTGTAAGTTTCAATTTTTTCTTACTTTTATGGC-3’
gRNA-TRP3-F	5’-AAAGGTCTCTGATCATCAAGCGTGCCTCTCCATCGGTTTTAGAGCTAGAAATAGCAAGT-3’
gRNA-TRP3-R	5’-AAAGGTCTCTAAACTGATACAGTCTCTTGAAATGCGATCATTTATCTTTCACTGCGGAG-3’
Doner-TRP3-F	5’-CATCGATTCCGTTTTGCTAACAAATAGCACTCAGCATCCTGCATAAAATTGGTATAAGATGCATGAAGGCTATCACGGTA-3’
Doner-TRP3-R	5’-TTTATATATGCGTAGTAATCACTCGGCAATGTGGAATTGTTACCGTGATAGCCTTCATGCATCTTATACCAATTTTATGC-3’
huan-TDH2-3-F	5’-AAGTACCACTGAGCTAGATCAGAGGGTGGTAAATGAAGTGTA-3’
huan-TDH2-3-R	5’-GAACTTGAAAAACTACGAATTTTGTTTTGTTTGTTTGTGTGATGAATTTAATTTGAAA-3’
huan-ACT1-3-F	5’-GAACTTGAAAAACTACGAATTTTGTTTTGTTTGTTTGTGTGATGAATTTAATTTGAAA-3’
huan-ACT1-3-R	5’-AAAACTACGAATTGTTAATTCAGTAAATTTTCGATCTTGGGAAGAAAAAAGCA-3’
huan-PDC1-3-F	5’-TACCACTGAGAGGGTAGCCTCCCCATAACAT-3’
huan-PDC1-3-R	5’-ACTACGAATTTTGATTGATTTGACTGTGTTATTTTGCG-3’
huan-1-5-TDH3-3-F	5’-AGACCGTTTGTTTGTTTATGTGTGTTTATTCGAAACT-3’
huan-1-6-TDH3-3-R	5’-GGAGGCTACCCTATAAAAAACACGCTTTTTCAGTTCGAG-3’
huan-1-6-PDC1-3-F	5’- TGTTTTTTATAGGGTAGCCTCCCCATAACATAA -3’
huan-1-6-PDC1-3-R	5’-ACTACGAATTTTGATTGATTTGACTGTGTTATTTTGCG -3’
huan-2-6-TPI1-3-F	5’-GACCGTTTTAGTTTATGTATGTGTTTTTTGTAGTTATAGATTTAAGCA -3’
huan-2-7-TPI1-3-R	5’-ACCCTCTGATCTAGTGTTTAAAGATTACGGATATTTAACTTACTTAGAATAATGCC-3’
huan-2-7-TDH2-3-F	5’-ATCTTTAAACACTAGATCAGAGGGTGGTAAATGAAGT -3’
huan-2-7-TDH2-3-R	5’-CTTGAAAAACTACGAATTTTGTTTTGTTTGTTTGTGTGATGAATT-3’
huan-1-5-TDH3-3-R	5’-GTGTGTCTTATATAAAAAACACGCTTTTTCAGTTCGAGT -3’
huan-1-5-ACT1-3-F	5’-AGCGTGTTTTTTATATAAGACACACGCGAGAACATATATAC-3’
huan-1-5-ACT1-3-R	5’-GAAAAACTACGAATTGTTAATTCAGTAAATTTTCGATCTTGGGAAG -3’
huan-1-7-TDH3-3-R	5’-CACCCTCTGATCTAGATAAAAAACACGCTTTTTCAGTTCGAGT -3’
huan-1-7-TDH2-3-F	5’-GTTTTTTATCTAGATCAGAGGGTGGTAAATGAAGT-3’
huan-1-7-TDH2-3-R	5’-AACTTGAAAAACTACGAATTTTGTTTTGTTTGTTTGTGTGAT

	GAATT -3’
huan-2-6-TPI1-3-R	5’-AGGCTACCCTTGTTTAAAGATTACGGATATTTAACTTACTTAGAATAATGC-3’
huan-2-6-PDC1-3-F	5’-ATCTTTAAACAAGGGTAGCCTCCCCATAACATAAA-3’
huan-2-6-PDC1-3-R	5’-AACTACGAATTTTGATTGATTTGACTGTGTTATTTTGCG -3’
PDC1-gRNA-F	5’-AAAGGTCTCTGATCAGCTGACTTGATTTTGTCTGTGTTTTAGAGCTAGAAATAGCAAGT-3’
PDC1-gRNA-R	5’-AAAGGTCTCTAAACTATAAGACTTGAGAGATACCGGATCATTTATCTTTCACTGCGGAG-3’
PDC5-gRNA-F	5’-AAAGGTCTCTGATCATTGACTTGAACGTCCCAGCCGTTTTAGAGCTAGAAATAGCAAGT-3’
PDC5-gRNA-R	5’-AAAGGTCTCTAAACTATAGAGCGCCGACTGTGAAAGATCATTTATCTTTCACTGCGGAG-3’
PDC6-gRNA-F	5’-AAAGGTCTCTGATCATGATTCATGGGCCTCACGCAGTTTTAGAGCTAGAAATAGCAAGT-3’
PDC6-gRNA-R	5’-AAAGGTCTCTAAACTATGGACCCCCACAACACCTGGATCATTTATCTTTCACTGCGGAG-3’
Donor-PDC1-F	5’-TCTCAATTATTATTTTCTACTCATAACCTCACGCAAAATAACACAGTCAAATCAATCAAAGCGATTTAATCTCTAATTAT-3’
Donor-PDC1-R	5’-TATTTTTCGTTACATAAAAATGCTTATAAACTTTAACTAATAATTAGAGATTAAATCGCTTTGATTGATTTGACTGTGT-3’
Donor-PDC5-F	5’-ACTTATTTCACATAATCAATCTCAAAGAGAACAACACAATACAATAACAAGAAGAACAAAGCTAATTAACATAAAACTCA -3’
Donor-PDC5-R	5’-ATAACCTTCAAAAGTAAAAAAATACACAAACGTTGAATCATGAGTTTTATGTTAATTAGCTTTGTTCTTCTTGTTATTGT-3’
Donor-PDC6-F	5’-TTTTATATACAGTATAAATAAAAAACCCACGTAATATAGCAAAAACATATTGCCAACAAAGCCATTAGTAGTGTACTCAA-3’
Donor-PDC6-R	5’-TCAAACTGTGTAAGTTTATTTATTTGCAACAATAATTCGTTTGAGTACACTACTAATGGCTTTGTTGGCAATATGTTTTT-3’

Table S2 Comparison of MP titer and specific productivity of different engineered strains

Strain	MP Titer (mg/L)	Max OD~600~	Specific Productivity (mg/g DCW·L)
BYL-1 (Control)	3.9 ± 0.3	12.5 ± 0.8	1.04 ± 0.08
BYL-8 (*ARO4*^{K229L})	13.4 ± 0.8	11.2 ± 0.6	3.99 ± 0.25
BYL-15 (△*TRP3*+△*ARO7*)	32.3 ± 1.5	10.7 ± 0.5	10.1 ± 0.5
BYL-26 (*IDP1p-Bbxfp*)	41.9 ± 1.9	9.8 ± 0.4	14.2 ± 0.7
BYL-49 (Optimized)	68.6 ± 2.5	10.1 ± 0.6	22.6 ± 1.4

Table S3 Genotype and copy numbers information of the plasmids used in our work

Plasmid	Type	Estimated Copy Number	Selection Marker	Key Features
pSPGM1	High-copy (2μ origin)	20–50 copies/cell	*URA3*	Used for *Ubic* and *Bsmt* co-expression (e.g., BYL-1 to BYL-3).
pRS425	High-copy (2μ origin)	20–50 copies/cell	*LEU2*	Employed for *Bbxfp* expression (e.g., BYL-25 to BYL-31).
pIYC04	Low-copy (CEN/ARS)	1–5 copies/cell	*HIS3*	Used for CRISPR/Cas9 knockouts (e.g., △*ARO7*/△*TRP3*).
